# Deficits in nominal reference identify thought disordered speech in a narrative production task

**DOI:** 10.1371/journal.pone.0201545

**Published:** 2018-08-07

**Authors:** Gabriel Sevilla, Joana Rosselló, Raymond Salvador, Salvador Sarró, Laura López-Araquistain, Edith Pomarol-Clotet, Wolfram Hinzen

**Affiliations:** 1 Grammar & Cognition Lab, Department of Translation and Language Sciences, Universitat Pompeu Fabra, Barcelona, Spain; 2 Grammar & Cognition Lab, Department of Catalan Philology and General Linguistics, Universitat de Barcelona, Barcelona, Spain; 3 FIDMAG Germanes Hospitalàries Research Foundation, Barcelona, Spain; 4 CIBERSAM (Centro de Investigación en Biomedicina en Red en Salud Mental), Barcelona, Spain; 5 Benito Menni Complex Assistencial en Salut Mental, Sant Boi de Llobregat, Barcelona, Spain; 6 Catalan Institute for Advanced Studies and Research (ICREA), Barcelona, Spain; Department of Psychiatry and Neuropsychology, Maastricht University Medical Center, NETHERLANDS

## Abstract

Formal thought disorder (TD) is a neuropathology manifest in formal language dysfunction, but few behavioural linguistic studies exist. These have highlighted problems in the domain of semantics and more specifically of reference. Here we aimed for a more complete and systematic linguistic model of TD, focused on (i) a more in-depth analysis of anomalies of reference as depending on the grammatical construction type in which they occur, and (ii) measures of formal grammatical complexity and errors. Narrative speech obtained from 40 patients with schizophrenia, 20 with TD and 20 without, and from 14 healthy controls matched on pre-morbid IQ, was rated blindly. Results showed that of 10 linguistic variables annotated, 4 showed significant differences between groups, including the two patient groups. These all concerned mis-uses of noun phrases (NPs) for purposes of reference, but showed sensitivity to how NPs were classed: definite and pronominal forms of reference were more affected than indefinite and non-pronominal (lexical) NPs. None of the measures of formal grammatical complexity and errors distinguished groups. We conclude that TD exhibits a specific and differentiated linguistic profile, which can illuminate TD neuro-cognitively and inform future neuroimaging studies, and can have clinical utility as a linguistic biomarker.

## Introduction

Formal thought disorder (TD) is a neuropathology clinically manifest in formal language dysfunction: expressive language is disorganized not at the level of the content of thought (*what* is expressed, as in the case of a delusion like ‘I have 1,000 children’), but its form (*how* it is said, i.e. its organization). It is a key symptom of schizophrenia according to the DSM-5, though not found in all patients with schizophrenia and not confined to this diagnosis. Functional neuroimaging studies of TD point to anomalies in language and speech processing regions [1]. Yet as reviewed below, very few behavioral linguistic studies of the formal aspects of thought disordered speech exist, especially in language production and in the context of contemporary linguistic theory. Rating scales like the *Scale for the Assessment of Thought*, *Language*, *and Communication* (TLC) [2] characterize TD through such terms as derailment, illogicality, tangentiality, or poverty of content. But it is not clear how (or even whether) these clinical descriptors map onto specific linguistic variables. Our aim here was to contribute to a more complete linguistic model of TD, which profiles TD at the level of its formal linguistic organization through objective variables. Such a profile can contribute to the cognitive neuropsychology of TD. Moreover, across different cognitive disorders, the potential role of language as a clinical marker has been emphasized based on evidence for its role in prediction, diagnosis, and tracking the disease process involved, including in schizophrenia [3–9], autism spectrum disorders [10], depression [11], Huntington’s disease [12], and Alzheimer’s [13].

Bleuler [14] coined the term ‘loose associations’ inspired by the associationist psychology of his time, which in a contemporary neuropsychological context conceptually connects to the notion of an increased ‘spread of activation’ across a semantic memory network, in which words are organized according to their lexical meanings [15–17]. In the generation of natural speech, however, words associate with each other not merely through statistical co-occurrence patterns or their lexical meanings, but by means of grammatical relations as well. It is only by virtue of these relations that utterances can be productively generated and express full thoughts with the referential meanings they carry in normal discourse. In line with this, Kuperberg [18] drew attention to ‘two streams of processing, one drawing upon semantic relationships within semantic memory and the other involving the use of combinatorial mechanisms to build propositional meaning’, and a potential shift in the ‘dynamic balance’ between these in schizophrenic speech. This hypothesis underlines the need for profiling linguistic dysfunction in TD at a structural or grammatical level, which we attempted here.

### Previous linguistic studies of spontaneous speech in TD

In unselected patients with schizophrenia, a number of previous linguistic studies have documented a pattern of reduced syntactic complexity and increased errors in spontaneous speech as compared with healthy controls [3,4,19–21]. These studies did not target TD, however, and there is some positive evidence that syntactic errors in linguistic production may characterize patients with SZ generally [22,23]. Sensitivity to errors of syntax specific to TD in speech perception have been identified as well, however [24], though a recent study [25] identified problems in detecting syntactic but not semantic anomalies in SZ at large, which did not correlate with the Thought and Language Index [26] as a measure of TD. Impairments in the semantic comprehension of syntactically complex expressions have also been reported [27], with some evidence of an association with TD [28].

Evidence from a linguistic case study comparing spontaneous speech in patients with TD (N = 6) to others without TD has pointed to a semantic-level anomaly specific to TD, identifiable at the single-sentence level [22]. The semantic deficit in question spared naming, i.e. lexical level meaning (see also [22,23,28–30]), hence implicating the codification of meaning at a grammatical rather than merely lexical level. All linguistic meaning in normal speech however also crosses the single-sentence level, insofar as every utterance contains noun phrases (NPs) within it, e.g. *the man who bought a hat*, *that cat*, or *she*, which serve to identify a particular man, hat, cat and female person, respectively. While utterances are fast-fading events, the referents in the world identified by NPs in it outlast any such utterance, being available for further referencing from the same or other speakers later on.

A number of linguistic studies have addressed this aspect of language in the context of TD from a communication- or discourse-theoretic perspective [31–35]. The notions of ‘unclear’ or ‘incompetent’ references have been highlighted in several of these studies [31–34]. This notion has not been systematically studied in its linguistic substrate, however. Reference is not a univocal notion in language, but there is a whole range of grammatically distinct ways in which NPs can establish reference to objects in the world. None of these ways is lexical only, insofar as a word in isolation, like *man*, only has general meaning, capturing a class of things: it cannot as such refer to a particular man, or *the man I saw yesterday*. Reference is a function of full NPs as embedded in sentences used in utterances on an occasion of language use. It is a grammatical phenomenon in this sense, which co-varies in its various forms with specific patterns of grammatical complexity and their functions [36,37]. ‘Definite’ NPs, in particular, such as *the taxi driver*, tend to be anaphoric, i.e. picking up on specific objects referenced in discourse before, while the function of a typical use of indefinites such as *a man* is to introduce a new object into the discourse. ‘Indefinite’ NPs, on the other hand, have no requirement of specificity, as in *doctors* *are on strike*, or *I have* *a doctor*. Finally, NPs can lack descriptive content altogether, being purely grammatical in nature, as in pronouns like *he*, which necessarily lack a lexical description: *he man* would be ungrammatical).

Without referencing, language would not express thought and could not have the real-world content that it does and that corresponds to our notion of truth. Since NPs link semantic memory (through their lexical basis) and discourse, via reference and grammar, NPs could be key to understanding the language of TD. Only when NPs are embedded under verb phrases (VPs) and VPs are embedded in sentences, however, can propositional meanings arise in which events are referenced in which objects and persons take part, and facts can be stated. Meaning at this global grammatical level depends on the specific forms of grammatical complexity involved. If sentences contain embedded clauses, as in *She thought that he wanted to eat her*, where *he wanted to eat her* is a clause embedded in another (i.e. the sentence as a whole), a particular kind of meaning is *ipso facto* encoded as well: the speaker expresses a thought about a thought of another person, specifying how that person represents the world. Sentences with this kind of meta-representational complexity are critical to reasoning about mental states and rationalizing actions that people make based on such states, but little or nothing is known on how they pattern in the language of TD, though previous evidence suggests that they are underrepresented in schizophrenic speech at large [4,19,21]. However, in some of these studies (e.g. [21]), complex and heterogeneous indices of ‘syntactic complexity’ are generated from sub-variables which combine what are linguistically very different phenomena, e.g. coordinated, subordinated, and relative clauses, along with passives and adjunct clauses. We therefore targeted embedded clauses separately here.

## Aims and hypotheses

We sought to contribute to a more complete linguistic model of the language of TD by determining whether a rater blind to diagnosis, using 10 purely linguistic variables preselected, could distinguish three groups based on their spontaneous speech in a narrative, impersonal task: patients with schizophrenia with and without TD, and healthy controls matched on estimated pre-morbid IQ. The choice of variables was driven by previous research and theoretical hypotheses, on the one hand, and an aim for more systematicity, on the other. Given prior evidence for a dysfunction of reference in TD, we firstly targeted the use of NPs using linguistically independently motivated sub-classifications of NP types in which such mis-uses occur. Our expectation specifically was that anomalies in the use of definite forms of reference would associate with TD more than indefinite ones, and pronouns more than all types of lexical NPs. This kind of pattern, if found, would provide us with a linguistic index of referencing problems in TD and how these are linked to grammar. Secondly, we selected objective and quantifiable linguistic indices of the formal grammatical complexity of sentences, namely embedded clauses as motivated above, and the number of grammatical dependents within a given utterance. We hypothesized that embedded clauses would be under-represented in patients with schizophrenia, and that also the number of dependents per utterance would generally be lower as compared to controls, without either difference being specific to TD [4,19,21]. We further hypothesized that no differences specific to TD might show in the domain of purely formal linguistic measures such as formal syntactic errors (e.g. word order or agreement violations). This was based on some evidence that semantic-level anomalies, either at the single-sentence level [22] or the level of reference in discourse [31–33], can distinguish patients with TD in speech production, while formal-syntactic errors affect patients with schizophrenia generally [22,23,38]; but also on theoretical grounds, insofar as TD is likely to map onto linguistic variables that affect the organization of meaning in language and hence the thinking expressed in speech, rather than being purely formal dimensions of language. Finally, we included lexical-level variables, motivated by both systematicity as linguistically motivated and previous literature for anomalies at this level in TD [39].

## Materials and methods

### Participants

20 patients with SZ and TD (SZ+TD) and 20 with SZ and without TD (SZ-TD) were recruited from the Hospital Benito Menni CASM, Sant Boi. All patients met DSM-4 diagnostic criteria. TD was assessed with the Thought, Language and Communication (TLC) scale [2], and patients were included in the TD group based on a total score of >1 (out of a total of 4). Of the 20 patients with TD, only 1 scored 2, while 13 scored 3 and 6 scored 4. 14 healthy controls (HC) were also recruited. Groups were matched on pre-morbid IQ as assessed with the TAP (Spanish NART; [40]). Patients were also assessed for PANSS total, positive and negative scores, CGI (Clinical global impressions) [41], and GAF (Global assessment of functioning) [42]. All participants were native Spanish-Catalan speakers. Clinical data are summarised in Table 1.

**Table 1:** Socio-demographic and clinical features of groups.

	Controls N = 14	Non TD N = 20	TD N = 20	Statistical test	p value
**Age (years)**	39.6 (10.83)	41.35 (8.99)	41.21 (12.48)	F = 0.160	0.853
**Sex (male/female) (N)**	8/6	12/8	12/8	X^2^ = 0.035	0.983
**IQ**	103.15 (14.92)	94.95 (18.35)	83.15 (10.74)	F = 7.426	<0.01
**IQ patients**		94.95 (18.35)	83.15 (10.74)	t = 2.483	0.019
**TAP**	100.14 (7.82)	99.85 (11.06)	97.60 (7.02)	F = 0.466	0.63
**Age of onset (years)**	-	22.05 (4.92)	18.33 (2.85)	t = 2.886	<0.01
**Duration of illness**	-	17.55 (9.90)	24.00 (9.63)	t = -2.032	0.05
**PANSS total**	-	66.55 (18.92)	85.40 (16.11)	t = -3.393	<0.01
**Positive syndrome**	-	13.70 (6.28)	17.25 (6.11)	t = -2.184	0.035
**Negative syndrome**	-	15.60 (5.45)	20.85 (6.18)	t = -3.849	<0.01
**Disorganised syndrome**	-	7.05 (2.19)	13.00 (2.62)	t = -2.533	0.016
**TLC**	-	0.08 (0.18)	3.15 (0.49)	t = -26.318	<0.01
**CGI**	-	4.05 (1.68)	5.10 (1.41)	t = -2.111	0.042
**GAF**	-	45.33 (14.82)	35.17 (10.50)	t = 2.302	0.028
**Antipsychotics First Generation (N)**	-	2	0		
**Antipsychotics Second Generation (N)**	-	17	10		
**Antipsychotics Combination First & Second Generation (N)**	-	1	10		
**Dose eq Chlorpromazine in mg**	-	624.57 (317.32)	1072.05 (595.45)	t = -2.852	<0.01

Values are stated as means with standard deviations unless otherwise indicated. *Abbreviations*: TAP: Test de Acentuación de Palabras (premorbid IQ); TLC: Scale for the Assessment of Thought, Language, and Communication; CGI: Clinical global impressions; GAF: Global assessment of functioning.

As can be seen in Table 1, all patients had drug treatment, but there was a significant difference in the amounts. Because of this, post hoc tests were performed to assess the potential confounding effect of the amount of medication (equivalents of chlorpromazine) on the linguistic variables that showed significant between group differences (see Results section).

This study (PR-2015-17) was conducted with the approval of the responsible ethical committee, CEIC, Comité de Ética en la Investigación Clínica, Hermanas Hospitalarias. The participants in the study were referred by their reference therapists, who knew the purpose of the study and who considered that the subjects could decide whether to participate or not; none of the participants were legally incapacitated. Each subject was subsequently evaluated by a member of the research team, who explained the study, answered possible questions or doubts and collected written informed consent. The authors assert that all procedures contributing to this work comply with the ethical standards of the relevant national and institutional committees on human experimentation and with the Helsinki Declaration of 1975, as revised in 2008.

### Procedure

All participants were asked to narrate a fairytale of their choice. Narrative requires introducing story characters and then tracing them throughout the storyline as events and actions unfold. As these events happen because of what protagonists believe or desire, mental states need to be referenced, which often requires using specific linguistic structures, e.g. *She thought that he wanted to eat her*, which are sentences in which the embedded clause (*he wanted to eat her*) figures. In different disorders, narrative has been shown to make speech difficulties manifest even where standardised language tests may fail do so [43–45].

Sessions were videotaped and rated for TD by two trained clinicians. TD was rated based on videos (by EP, SS, and LL), but these raters were not involved in transcription or annotation. The first author of this study (GS), who was never in contact with the participants and blind to diagnosis or level of TD, transcribed and annotated the narrations of all participants based on their audios only, to avoid any visual bias regarding the participants’ clinical condition. Transcriptions were carried out with the transcription software CLAN [46]. During the annotation process, a consensus-based approach was used in which the first, second and last authors met weekly to discuss all annotations and address questionable cases, which were resolved by agreement in all cases. Post hoc, these consensus ratings were checked for inter-rater reliability by involving a further rater (Clara Soberats) who had not been involved in this study until this point, had no knowledge of its aims, and was also blind to group membership. This independent rater recoded a random selection of 20% (n = 11) of the transcribed narratives. Reliability was calculated for all ten variables based on point-to-point agreement using the formula: the number of times the two ratings agreed ÷ the number of times the two coders agreed + disagreed.

### Linguistic variables and annotation

Ten linguistic variables were annotated, starting from anomalies in the referential use of 3^rd^ Person NPs, subclassified according to the type of NP in which they occurred: *pronouns* (e.g. *she*), NPs that involve *lexical nouns* (e.g. *the* *girl*, *a* *man*), NPs that are *definite* (e.g. *her grandmother*, *this girl*, *Red Riding Hood*, *she*), and NPs that are *indefinite* (e.g. *some girl*, *a man*, *food*, *men*). Only anomalies in NPs in grammatical 3^rd^ Person were annotated, since due to the impersonal nature of the task, 1^st^ and 2^nd^ Person would be largely extraneous to it and also unlikely to occur. Errors of content or narrative correctness (e.g. *The girl ate the wolf*, when it clearly can only be the other way around), were *not* annotated as referential anomalies. A typical example of a referential anomaly would be that a pronoun such as *he* is used but the referent cannot be made out, or that a definite NP is used when an indefinite is expected, or vice versa. Finally, we annotated mistaken lexical choices (paraphasias, e.g. *adjacentment* for *wall* or *park* instead of *forest*), and violations of semantic selectional restrictions (e.g. *The pond fell in the front doorway*, where the lexical meaning of the words involved tells us that a *pond*, say, cannot *fall*).

Apart from the five variables capturing referential anomalies (1. Definite NPs, 2. Indefinite NPs, 3. Pronouns, 4. Lexical NPs, 5. 3^rd^ Person NPs), and the lexical-level variables, (6. Paraphasia, and 7. Violations of semantic selectional restrictions), in the domain of sentence-level grammatical complexity and integrity we computed 8. the number of embedded (complement) clauses, and 9. the number of grammatical dependents, which were counted for each utterance. Dependents were counted by identifying each utterance’s ‘head’, usually the verb, around which other phrases are grouped, e.g. *the girl* and *the grandmother* are dependents of *visited* in *The girl visited the grandmother*. Finally, we counted: 10. Formal grammatical errors, i.e. violations of grammatical well-formedness conditions as detected on a single-sentence level. S1 Appendix summarizes this annotation scheme with definitions and examples.

### Statistical analysis

Two different statistical models were fitted to evaluate potential group differences in the scores of the 10 linguistic variables. For those variables registering unusual events usually involving a small number of occurrences (all but the number of grammatical dependents), negative binomial generalised linear models were usually fitted. Such models are equivalent to Poisson models but are more flexible, allowing for over dispersion. An offset term containing the total number of words was added to the models to account for dissimilar speech lengths. Alternatively, normal general linear models were applied for the number of grammatical dependents. Finally, due to the lack of anomalies in indefinites in the group of controls, a Fisher exact test had to be used with this variable. Previously, individual scores had to be binarised (i.e. presence or absence of anomalies in indefinites).

Each one of the models fitted reported an ANOVA like significance value that indicated if there was any difference between groups. A false discovery rate (FDR) correction for multiple comparisons [47] was applied to the 10 models. For those linguistic variables remaining significant after FDR correction, pairwise comparisons were carried out by means of linear contrasts to know the differing groups. A second FDR correction was applied to these pairwise comparisons. The analysis was carried out in R (https://www.R-project.org/) [48].

## Results

Of the 10 linguistic variables, 4 were significant after FDR correction. These were anomalies in definite NPs, pronouns, 3^rd^ person NPs, and paraphasias (see Table 2). When linear contrasts were carried out on these significant variables, the majority of pairwise differences were also found significant. The exceptions were, for the non-TD schizophrenia group versus the control group contrast, in the pronouns and in the paraphasias (see Table 3).

**Table 2:** General statistical significance for possible between group differences in the linguistic variables.

Linguistic variable	Model type	statistic	p-value	FDR corrected p
Definite	NegBin-GLM	X^2^ = 16.01	0.0003	0.0016
Indefinite	Fisher exact	None	0.0531	0.1063
Pronoun	NegBin-GLM	X^2^ = 13.02	0.0014	0.0049
Noun	NegBin-GLM	X^2^ = 4.995	0.0822	0.1371
Complement clause	NegBin-GLM	X^2^ = 1.802	0.4062	0.4512
Semrestr	NegBin-GLM	X^2^ = 4.584	0.1013	0.1443
FGE	NegBin-GLM	X^2^ = 1.162	0.5594	0.5593
Person3	NegBin-GLM	X^2^ = 16.98	0.0002	0.0016
Paraphasia	NegBin-GLM	X^2^ = 10.06	0.0065	0.0163
Nofdeput	Normal-GLM	F = 1.053	0.3562	0.4452

*Abbreviations*: Semrestr: violation of semantic selectional restrictions, FGE: Formal grammatical errors, Nofdeput: Number of dependents per utterance.

**Table 3:** Results of the pairwise contrasts between groups for the four variables found to be significant in the general analyses.

Variable	Groups	Relative risk	Statistic	p-value	FDR p-value
Definite	SZ-TD vs. CONT	3.602	X^2^ = 5.285	0.021	0.0438
	SZ+TD vs. CONT	8.458	X^2^ = 15.44	8.4e-05	0.0005
	SZ+TD vs. SZ-TD	2.348	X^2^ = 4.268	0.0388	0.0465
Prono	SZ-TD vs. CONT	3.183	X^2^ = 3.077	0.0793	0.0865
	SZ+TD vs. CONT	9.335	X^2^ = 12.17	0.0004	0.0016
	SZ+TD vs. SZ-TD	2.933	X^2^ = 4.466	0.0345	0.0460
person3	SZ-TD vs. CONT	3.419	X^2^ = 4.983	0.0256	0.0438
	SZ+TD vs. CONT	8.533	X^2^ = 16.08	6.1e-05	0.0005
	SZ+TD vs. SZ-TD	2.496	X^2^ = 5.073	0.0242	0.0438
Paraph	SZ-TD vs. CONT	5.354	X^2^ = 2.353	0.1250	0.1250
	SZ+TD vs. CONT	16.67	X^2^ = 7.166	0.0074	0.0222
	SZ+TD vs. SZ-TD	3.114	X^2^ = 4.556	0.0328	0.0460

*Abbreviations*: CONT: Healthy controls. SZ +/- TD: participant with schizophrenia with or without TD.

**Fig 1:**
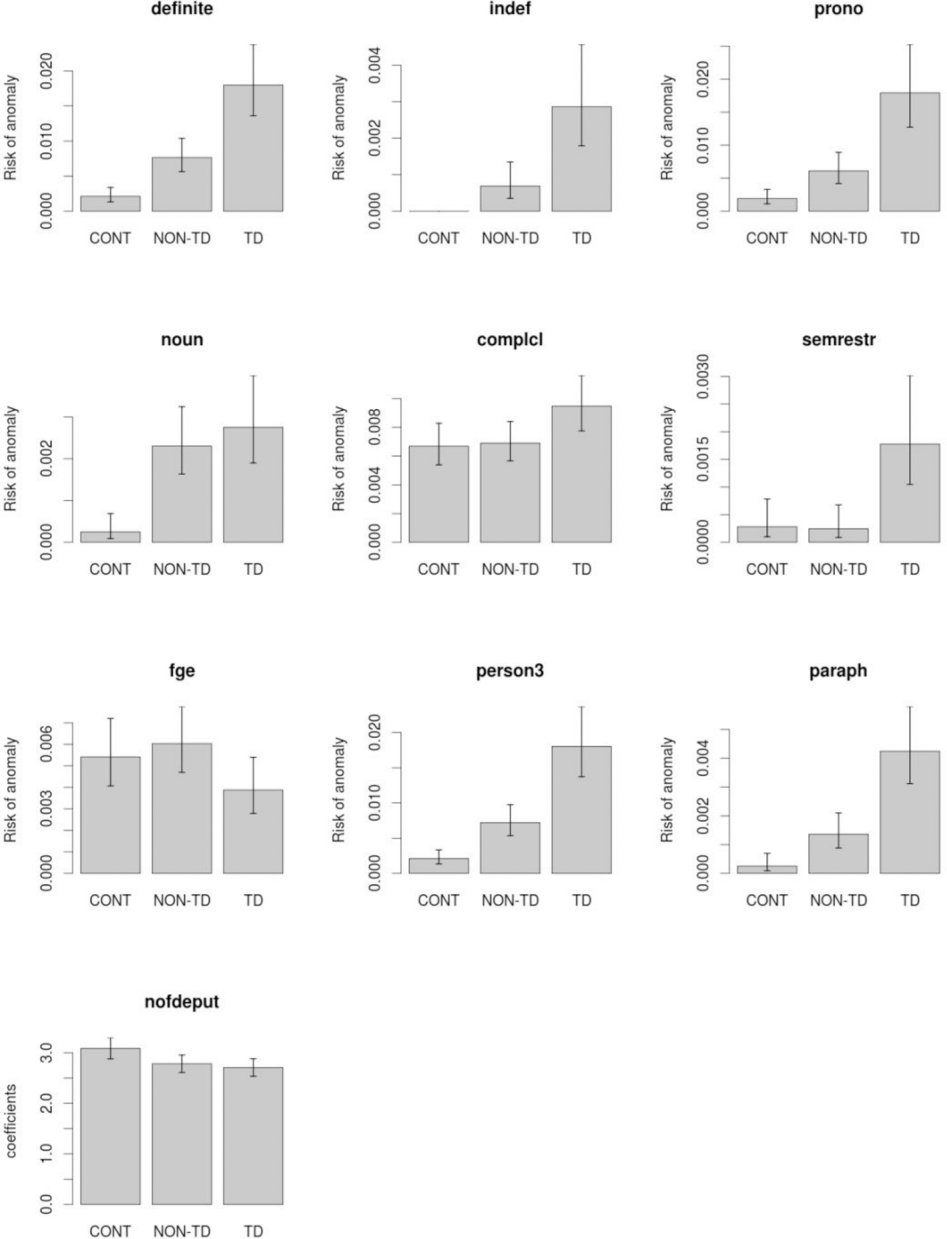
Estimates of risk of anomaly for the different variables and groups. Probabilities of occurrence of an anomaly are plotted for all variables and groups. Bars shown are 95% confidence intervals for the risk estimates. *Abbreviations*: indef: indefinites; prono: pronouns; complcl: complement clauses; semrestr: violation of semantic selectional restrictions; fge: formal grammatical errors; person3: 3^rd^ grammatical person; nofdeput: number of dependents per utterance; CONT: neurotypical controls; NON-TD: patients without thought disorder; TD: patients with thought disorder.

Fig 1 below shows the estimates of risk of anomaly (probability of occurrence of an anomalous word) for the different variables and groups. A gradual increase in risk from the control group to the non-TD group, and from the latter to the TD group is evident from the plots of the four significant variables. Indeed, the estimates of relative risk, which are the ratios of risk estimates between two groups, were clearly larger than 2.0 in all significant pairs (see Table 3) implying more than a two-fold increase in risk of anomaly between groups (indeed, relative risks for the td vs. controls (cnt) pair were larger than 10 in three of the significant variables).

To check for the potentially confounding effect of medication in these results, we fitted the same negative binomial models (used before to see if there were differences between groups), but considering the amount of medication instead of the group as the independent variable. Only patients were included. No effect of amount of medication on the relevant language variables was found: Definite: *p* = 0.4206, Pronoun: *p* = 0.377, Person3: *p* = 0.4504, Paraphasia: *p* = 0.8620.

Finally, the post hoc check on the reliability of the consensus rating by an independent rater revealed that agreement between both ratings in the cases of ‘Paraphasia’ and ‘Semantic Selectional Restrictions’ fell to 66,66% and 0%, respectively. This was partially due to the small number of instances in both cases (Paraphasia: independent rater agreed on 2 out of a total of 3 cases in the consensus rating; Semantic Selectional Restrictions: independent rater found 0 as against 1 instance in the consensus rating). The mean agreement for the remaining eight variables was 89,77% (range: 80%-100%) showing that reliability of coding was consistently in the high range.

## Discussion

The results demonstrate a differentiated profile in which some linguistic variables showing sensitivity to TD divide from others that do not. In particular, while all patients could generally perform the task of telling a fairytale from memory, referential anomalies in the use of NPs distinguished groups in all pairwise comparisons, with a gradual increase of risk of such anomalies from controls to patients without TD to patients with TD. This predicted result holds despite of the fact that telling a fairytale is a task that is semantically well-circumscribed, in the sense that a fixed set of characters must be referenced and tracked through the storyline. By contrast, speech in the TD group was neither formally more ungrammatical nor less complex grammatically than that of any of the comparator groups.

Results also confirmed our hypothesis that not all 3^rd^ Person NPs would behave the same: while anomalies in the use of definite and pronominal NPs distinguished groups including the two patient groups–with TDs exhibiting significantly more likelihood of such anomalies in both–this was not the case for the variables indefinite or noun (i.e. lexical/non-pronominal nominals). However, more work in larger samples and with more speech quantity is required to confirm that the deficits in TD seen to be specific to definite and pronominal NPs here do not extend to indefinite and lexical ones, or do so to a lesser extent.

Interestingly, and contrary to predictions, one measure of meaning at the (single) sentence-level but more rooted in aspects of *lexical* semantics, such as violations of semantic selectional restrictions (see [22]), also turned out to be non-significant. In the case of such a violation, it is the lexical meaning/concepts alone that tell us that a rule has been violated–we know from the meaning of *forest*, say, that a forest cannot be *drunk*. This is crucially different in the anomalies of reference that we report here, which *are* inherently related to context and discourse. If a patient says *a girl* but *the girl* would be expected, or mis-uses a pronoun, the problem does not lie at the lexical end, but in how a given lexical concept is referentially used via grammar. Given the link to context, a defect in this mechanism could relate to pragmatic impairments noted in FTD, interconnecting linguistic levels [49,50]. Another semantic-level variable, equally lexically based, was paraphasia, which did reveal significant differences between patients with TD and both controls and patients without TD. Paraphasias may relate to the referential function of language indirectly, insofar as reference in language is mediated by lexical description (concepts), and hence a correct lexical choice has to be made when a lexical concept is used as part of the NP. We speculate that a paraphasic deficit might thus be interpreted to indicate a deficit affecting reference in connected speech at its lexical end, particularly since this phenomenon has not been reported in lexical naming tasks.

The above result concerning NP types raises the theoretical question of what makes definite and pronominal NPs different from indefinite and non-pronominal types. An empirical difference between them lies in their respective mediation by lexical content. This is obvious for the case of pronouns when compared to NPs involving lexical nouns but can also be seen in comparing definites with indefinites. Thus, in the latter, the act of reference is largely rooted in the lexical concept entering the NP: looking for *food* or *a policeman*, say, is to look for anything that qualifies as a food or a policeman, respectively, i.e. satisfies the descriptive content of the lexical nominal, while no specific referent is required. This is different with both definites and pronouns, where lexical descriptions of the intended referent are either absent (in the case of pronouns), or need not be satisfied for the specific referent denoted to be picked out successfully in communication (as when a person referred to as *the policeman over there* turns out to actually be a *fireman*, but the intended referent is picked out and successfully conveyed nonetheless [51]). Moreover, a definite NP functioning anaphorically will typically re-identify an individual under an already used description, which is then dropped completely under continuation of the narrative (*I met* *a man* *this morning…*. *The man* *said …* *He**…*), suggesting a progression towards more grammaticalized forms of reference starting from more lexicalized ones, from indefinite NPs to definites and finally to pronouns. Our results about NP types in this sense suggest that in TD the system of reference in language is most challenged towards its grammatical end. This is also because when a referential anomaly was annotated in a definite NP, it was the NP as a whole that was anomalous, while its lexical part (the noun it contains) was typically normal. It has been suggested on independent linguistic grounds [36,37,52] that the spectrum of forms of reference available in language can be ordered in a hierarchy, with the upper (definite-specific) regions of the hierarchy involving more grammatical complexity than the lower (indefinite) ones. On these grounds as well, differential impairment in these upper regions as compared with the lower ones suggests a deficit in grammar-mediated forms of reference, which is also in line with the absence of impairment often noted in TD in lexical (e.g. picture-naming) tasks [15,22,28–30].

Our findings also update extant knowledge by not confirming evidence from studies of unselected patients with schizophrenia that have found reduced syntactic complexity and more syntactic errors in such patients, which in our terms would predict more formal grammatical errors, less complement clauses, or less number of dependents per utterance in the patient groups relative to controls [3,4,19,21,22,53]. Insofar as the latter two variables are indices of the formal complexity of thought expressed, there were no differences in such formal complexity between any of our groups. Problems of reference thus appear as a different dimension of language than can be measured by these other variables. Our results *are* consistent with the findings of discourse- and communication-based studies [31,54], where difficulties with pronouns and reference have been long noted. Our findings however suggest that this problem is specifically traceable to particular grammatical types of NPs (definites and pronouns) and their normal functions, of which the discourse-theoretic function of ‘cohesion’ is merely one.

This leads to a further question, what might explain the linguistic patterns found and to what extent they reveal a primary linguistic impairment. It seems undeniable that referentiality *is* inherent to normal linguistic functioning–the absence of referentiality, as in echolalia, would be a clinical phenomenon. Moreover, forms of reference available cross-linguistically co-vary with specific forms of grammatical complexity. Moreover, although reference as such is available nonverbally (as in index-finger pointing) and not specific to humans, such nonverbal forms of reference, in their declarative varieties, closely relate to language in human development [55,56], and are not found in the same forms in non-linguistic beings including monkeys and non-human primates [57,58]. It would follow from this reasoning that TD involves a language dysfunction by involving a distinctive disturbance of reference. Though other dimensions of language showed no group effects in the present study, particularly our measures of syntactic complexity and integrity (errors), a number of previous studies [24,28,59,60], but not others [22,23,25], have shown a relation between TD and syntax as well. The present study contributes to clarifying this contradictory situation by showing that for the linguistically more specific syntactic measures used here, no group differences are seen. Future work should corroborate the existence of syntactic impairments and their specificity to TD, as well raise this issue of specificity for other linguistic domains such as pragmatics [49,50].

Note that TD shares the feature of affecting reference with other neurodevelopmental and neurodegenerative disorders, where reference often transpires as a locus of vulnerability, though it disintegrates in different and pathology-specific ways [52]. This suggests the need to include the domain of language in neuropsychological models of TD, which have so far primarily linked TD to impairments in semantic memory [15,16,23,30,61–63], and executive functioning [64–67]. However, findings have been mixed in both cases [67,68], patients with TD typically underperform on a wide range of cognitive tasks [68], and there is evidence for a general association between IQ and TD [69–72]. Associations between linguistic deficits documented here and these other neuropsychological measures are an important desideratum for future work. As of now, the neurocognitive basis of reference has barely been explored (but see [73–76]), unlike the neurocognitive basis of semantic memory [77–79]. Our results also bear on the questions of what to look for (and not) in speech output investigated for its predictive or diagnostic role, and of how to design new clinical linguistic tools for the early detection of different forms of cognitive decline.

Limitations of this study include effects that are non-significant potentially due to the reduced discriminatory power arising from a small sample size. Moreover, although the present study covers the referential use of language and aspects of its grammatical complexity, language is a highly complex domain that involves multiple other dimensions besides the ones studied here, particularly including phonetic-acoustic and pragmatic ones. These interface with the ones studied here and need to be investigated to further build a language profile of productive speech in TD.

In summary, this study has provided evidence that under conditions of blind rating based on linguistic criteria, TD is identifiable through the (mis-) use of NPs and of some classes of NPs more than others, while other linguistic dimensions including clausal embedding, formal grammatical errors, and grammatical complexity, do not associate with TD. NPs are the main devices in language that embody its inherent referentiality: language relates to the world, carrying content. A linguistic dysfunction in grammar-mediated forms of reference, therefore, could illuminate the neurocognition of TD, and it calls for exploring its clinical utility as well.

## Supporting information

S1 Appendix List of variables, definitions and examples. NB: Examples of the first 7 variables illustrate anomalies.
(DOCX)

S1 Dataset Final dataset FIDMAG. *Abbreviations*: id: identity, group 0: controls, group 1: schizophrenia without thought disorder, group 2: schizophrenia with thought disorder, tap: test de acentuación de palabras (premorbid IQ), iq: intelligence quotient, fsiq: tap values rescaled to iq units, age: chronological age, tlc: scale for the assessment of thought, language, and communication, nutter: number of utterances, nwords: number of words, prono: pronominal noun phrase, noun: lexical noun phrase, definite: definite noun phrase, indef: indefinite noun phrase, person3: third grammatical person, paraph: paraphasia, semrestr: violation of semantic selectional restrictions, nofdeput: number of dependents per utterance, complcl: complement clause, fge: formal grammatical errors, eqp: equivalents of chlorpromazine.
(XLSX)

## References

[1] Wensing T, Cieslik EC, Müller VI, Hoffstaedter F, Eickhoff SB, Nickl-Jockschat T. Neural correlates of formal thought disorder: An activation likelihood estimation meta-analysis. Hum Brain Mapp. 2017;38(10):4946–65. 10.1002/hbm.23706 28653797 PMC5685170

[2] Andreasen NC. Thought, language, and communication disorders. I. Clinical assessment, definition of terms, and evaluation of their reliability. Arch Gen Psychiatry. 1979 11;36(12):1315–21. 496551 10.1001/archpsyc.1979.01780120045006

[3] Fraser WI, King KM, Thomas P, Kendell RE. The diagnosis of schizophrenia by language analysis. Br J Psychiatry. 1986;148:275–8. 3719219 10.1192/bjp.148.3.275

[4] Morice R, McNicol D. Language changes in schizophrenia: a limited replication. Schizophr Bull. 1986;12(2):239–51. 3715418 10.1093/schbul/12.2.239

[5] Bedi G, Carrillo F, Cecchi GA, Slezak DF, Sigman M, Mota NB, et al Automated analysis of free speech predicts psychosis onset in high-risk youths. NPJ Schizophr. 2015;1(1):15030. 27336038 10.1038/npjschz.2015.30 PMC4849456

[6] Bearden CE, Wu KN, Caplan R, Cannon TD. Thought disorder and communication deviance as predictors of outcome in youth at clinical high risk for psychosis. J Am Acad Child Adolesc Psychiatry. 2011;50(7):669–80. 10.1016/j.jaac.2011.03.021 21703494 PMC3124656

[7] Rosenstein M, Foltz PW, DeLisi LE, Elvevåg B. Language as a biomarker in those at high-risk for psychosis. Schizophr Res. 2015;165(2–3):249–50. 10.1016/j.schres.2015.04.023 25956631

[8] Brown M, Kuperberg GR. A Hierarchical Generative Framework of Language Processing: Linking Language Perception, Interpretation, and Production Abnormalities in Schizophrenia. Front Hum Neurosci. 2015;9(November):1–23. 10.3389/fnhum.2015.00001 26640435 PMC4661240

[9] Mota NB, Copelli M, Ribeiro S. Thought disorder measured as random speech structure classifies negative symptoms and schizophrenia diagnosis 6 months in advance. npj Schizophr. 2017;3(1):18. 28560264 10.1038/s41537-017-0019-3 PMC5441540

[10] Kuhl PK, Coffey-Corina S, Padden D, Munson J, Estes A, Dawson G. Brain Responses to Words in 2-Year-Olds with Autism Predict Developmental Outcomes at Age 6. PLoS One. 2013;8(5). 10.1371/journal.pone.0064967 PMC3666972 23734230

[11] Fineberg SK, Leavitt J, Deutsch-Link S, Dealy S, Landry CD, Pirruccio K, et al Self-reference in psychosis and depression: A language marker of illness. Psychol Med. 2016;46(12):2605–15. 10.1017/S0033291716001215 27353541 PMC7944937

[12] Hinzen W, Rosselló J, Morey C, Camara E, Garcia-Gorro C, Salvador R, et al A systematic linguistic profile of spontaneous narrative speech in pre-symptomatic and early stage Huntington’s disease. Cortex. 2017;00(0). 10.1016/j.cortex.2017.07.022 PMC5845634 28859906

[13] Ahmed S, Haigh A-MF, de Jager CA, Garrard P. Connected speech as a marker of disease progression in autopsy-proven Alzheimer’s disease. Brain. 2013;136(12):3727–37. 24142144 10.1093/brain/awt269 PMC3859216

[14] Bleuler E. Dementia praecox, oder Gruppe der Schizophrenien [Dementia Praecox or the Group of Schizophrenias] Leipzig Franz Deuticke; 1911.

[15] Goldberg TE, Aloia MS, Gourovitch ML, Missar D, Pickar D, Weinberger DR. Cognitive substrates of thought disorder, I: The semantic system. Am J Psychiatry. 1998;155(12):1671–6. 10.1176/ajp.155.12.1671 9842774

[16] Aloia MS, Gourovitch ML, Missar D, Pickar D, Weinberger DR, Goldberg TE. Cognitive substrates of thought disorder, II: Specifying a candidate cognitive mechanism. Am J Psychiatry. 1998;155(12):1677–84. 10.1176/ajp.155.12.1677 9842775

[17] Pomarol-Clotet E, Oh TM, Laws KR, McKenna PJ. Semantic priming in schizophrenia: systematic review and meta-analysis. Br J Psychiatry. 2008 2;192(2):92–7. 10.1192/bjp.bp.106.032102 18245021

[18] Kuperberg GR. Language in schizophrenia Part 2: What can psycholinguistics bring to the study of schizophrenia…and vice versa? Lang Linguist Compass. 2010;4(8):590–604. 10.1111/j.1749-818X.2010.00217.x 20824153 PMC2932455

[19] Morice RD, Ingram JCL. Language Analysis in Schizophrenia: Diagnostic Implications. Aust New Zeal J Psychiatry. 1982 6 26;16(2):11–21. 10.3109/00048678209161186 6957177

[20] Thomas P, King K, Fraser WI, Kendell RE. Linguistic performance in schizophrenia: a comparison of acute and chronic patients. Br J Psychiatry. 1990;156:204–10. 2317624 10.1192/bjp.156.2.204

[21] Tavano A, Sponda S, Fabbro F, Perlini C, Rambaldelli G, Ferro A, et al Specific linguistic and pragmatic deficits in Italian patients with schizophrenia. Schizophr Res. 2008;102(1–3):53–62. 10.1016/j.schres.2008.02.008 18396387

[22] Oh T, McCarthy R, McKenna P. Is There a Schizophasia? A Study Applying the Single Case Approach to Formal Thought Disorder in Schizophrenia. Neurocase. 2002 6;8(3):233–44. 10.1093/neucas/8.3.233 12119320

[23] Stirling J, Hellewell J, Blakey A, Deakin W. Thought disorder in schizophrenia is associated with both executive dysfunction and circumscribed impairments in semantic function. Psychol Med. 2006 4;36(4):475–84. 10.1017/S0033291705006884 16403241

[24] Kuperberg GR, Mcguire PK, David AS. Reduced Sensitivity to Linguistic Context in Schizophrenic Thought Disorder: Evidence From On-Line Monitoring for Words in Linguistically Anomalous Sentences. J Abnorm Psychol. 1998;107(3):423–34. 9715577 10.1037//0021-843x.107.3.423

[25] Moro A, Bambini V, Bosia M, Anselmetti S, Riccaboni R, Cappa SF, et al Detecting syntactic and semantic anomalies in schizophrenia. Neuropsychologia. 2015;79:147–57. 10.1016/j.neuropsychologia.2015.10.030 26519554

[26] Liddle PF, Ngan ETC, Caissie SL, Anderson CM, Bates AT, Quested DJ, et al Thought and Language Index: an instrument for assessing thought and language in schizophrenia. Br J Psychiatry. 2002 10;181:326–30. 12356660 10.1192/bjp.181.4.326

[27] Condray R, Steinhauer SR, van Kammen DP, Kasparek A. The language system in schizophrenia: Effects of capacity and linguistic structure. Schizophr Bull. 2002;28(3):475–90. 12645679 10.1093/oxfordjournals.schbul.a006955

[28] Tan EJ, Yelland GW, Rossell SL. Characterising receptive language processing in schizophrenia using word and sentence tasks. Cogn Neuropsychiatry. 2016 1;21(1):14–31. 10.1080/13546805.2015.1121866 27031118

[29] Walenski M, Weickert TW, Maloof CJ, Michael T. Grammatical processing in schizophrenia: evidence from morphology. Neuropsychologia. 2010;48(1):262–9. 10.1016/j.neuropsychologia.2009.09.012 19766129 PMC2794971

[30] Barrera a, McKenna PJ, Berrios GE. Formal thought disorder in schizophrenia: an executive or a semantic deficit? Psychol Med. 2005 1;35(1):121–32. 15842035 10.1017/s003329170400279x

[31] Rochester S, Martin JR. Crazy Talk: A Study of the Discourse of Schizophrenic Speakers New York: Plenum Press; 1979.

[32] Harvey PD. Speech competence in manic and schizophrenic psychoses: the association between clinically rated thought disorder and cohesion and reference performance. J Abnorm Psychol. 1983;92(3):368–77. 6619412 10.1037//0021-843x.92.3.368

[33] Chaika E, Lambe RA. Cohesion in schizophrenic narratives, revisited. J Commun Disord. 1989;22(6):407–21. 2621258 10.1016/0021-9924(89)90034-8

[34] Barch D, Berenbaum H. Language Production and Thought Disorder in Schizophrenia. J Abnorm Psychol. 1996;105(1):81–8. 8666714 10.1037//0021-843x.105.1.81

[35] Docherty NM, Hall MJ, Gordinier SW, Cutting LP. Conceptual sequencing and disordered speech in schizophrenia. Schizophr Bull. 2000;26(3):723–35. 10993409 10.1093/oxfordjournals.schbul.a033489

[36] Hinzen W, Sheehan M. The philosophy of universal grammar Oxford Univ. Press; 2013.

[37] Martin T, Hinzen W. The grammar of the essential indexical. Lingua. 2014;148:95–117.

[38] Morice R, McNicol D. Language changes in schizophrenia: a limited replication. Schizophr Bull. 1986;12(2):239–51. 3715418 10.1093/schbul/12.2.239

[39] Covington MA, He C, Brown C, Nac L, Mcclain JT, Sirmon B, et al Schizophrenia and the structure of language: The linguist ‘s view. Schizophr Res. 2005;77:85–98. 10.1016/j.schres.2005.01.016 16005388

[40] Gomar JJ, Ortiz-Gil J, McKenna PJ, Salvador R, Sans-Sansa B, Sarró S, et al Validation of the Word Accentuation Test (TAP) as a means of estimating premorbid IQ in Spanish speakers. Schizophr Res. 2011;128(1–3):175–6. 10.1016/j.schres.2010.11.016 21144711

[41] Guy W. ECDEU Assessment Manual for Psychopharmacology: Revised. ECDEU Assess Man. 1976;217–22.

[42] Spitzer RL, Gibbon M, Williams J, Endicott J. Global Assessment of Functioning (GAF) Scale In: L S B D, editors. Outcomes Assessment in Clinical Practice. Baltimore: Williams and Wilkins; 1996 p. 76–8.

[43] Norbury CF, Gemmell T, Paul R. Pragmatics abilities in narrative production: a cross-disorder comparison. J Child Lang. 2014;41(3):485–510. 10.1017/S030500091300007X 23632039

[44] Banney R, Harper-Hill K, Arnott WL. The Autism Diagnostic Observation Schedule and narrative assessment: Evidence for specific narrative impairments in autism spectrum disorders. Int J Speech Lang Pathol. 2015;17(2):159–71. 10.3109/17549507.2014.977348 25541740

[45] Zinken J, Blakemore C, Zinken K, Butler L, Skinner TC. Narrating psychological distress: Associations between cross-clausal integration and mental health difficulties. Appl Psycholinguist. 2011;32(2):263–74.

[46] MacWhinney B. The CHILDES Project: Tools for Analyzing Talk Mahwah, NJ: Lawrence Erlbaum Associates; 2000.

[47] Benjamini Y, Hochberg Y. Controlling the False Discovery Rate: A Practical and Powerful Approach to Multiple Testing. J R Stat Soc Ser B. 1995;57(1):289–300.

[48] R Core Team. R: A language and environment for statistical computing Vienna, Austria: R Foundation for Statistical Computing 2014.

[49] Bosia M, Arcara G, Buonocore M, Bechi M, Moro A, Cavallaro R, et al Communication in schizophrenia, between pragmatics, cognition, and social cognition. In 2016 p. 213–34.

[50] Salavera C, Puyuelo M, Antoňanzas JL, Teruel P. Semantics, pragmatics, and formal thought disorders in people with schizophrenia. Neuropsychiatr Dis Treat. 2013; 10.2147/NDT.S38676 PMC3573805 23430043

[51] Donnellan KS. Reference and Definite Descriptions. Philos Rev. 1966;75(3):281.

[52] Hinzen W. Reference across pathologies: A new linguistic lens on disorders of thought. Theor Linguist. 2017;43(3–4):169–232.

[53] Thomas P, King K, Fraser WI, Kendell RE. Linguistic performance in schizophrenia: a comparison of acute and chronic patients. Br J psychiatry J Ment Sci. 1990;156:204–10, 214–5. 10.1192/bjp.156.2.204 2317624

[54] Docherty NM, DeRosa M, Andreasen NC. Communication disturbances in schizophrenia and mania. Arch Gen Psychiatry. 1996 4;53(4):358–64. 8634014 10.1001/archpsyc.1996.01830040094014

[55] Iverson JM, Goldin-Meadow S. Gesture Paves the Way for Language Development. Psychol Sci. 2005 5 1;16(5):367–71. 10.1111/j.0956-7976.2005.01542.x 15869695

[56] Butterworth G. Pointing is the royal road to language for babies In: Kita S, editor. Pointing: where language, culture, and cognition meet. Lawrence Erlbaum Associates; 2008 p. 9–33.

[57] Tempelmann S, Kaminski J, Liebal K. When apes point the finger: Three great ape species fail to use a conspecific’s imperative pointing gesture. Interact Stud. 2013 1;14(1):7–23.

[58] Tomasello M, Call J. Thirty years of great ape gestures. Anim Cogn. 2018;(0123456789):1–9. 10.1007/s10071-017-1151-1 29468285 PMC6647417

[59] Walenski M, Weickert TW, Maloof CJ, Ullman MT. Grammatical processing in schizophrenia: Evidence from morphology. Neuropsychologia. 2010;48(1):262–9. 10.1016/j.neuropsychologia.2009.09.012 19766129 PMC2794971

[60] Faber R, Reichstein MB. Language dysfunction in schizophrenia. Br J Psychiatry. 1981;139:519–22. 7332856 10.1192/bjp.139.6.519

[61] Tamlyn D, McKenna PJ, Mortimer a. M, Lund CE, Hammond S, Baddeley a. D. Memory impairment in schizophrenia: its extent, affiliations and neuropsychological character. Psychol Med. 2009 7;22(01):101. 10.1017/s0033291700032773 1349439

[62] Kreher D a, Holcomb PJ, Goff D, Kuperberg GR. Neural evidence for faster and further automatic spreading activation in schizophrenic thought disorder. Schizophr Bull. 2008 5;34(3):473–82. 10.1093/schbul/sbm108 17905785 PMC2632424

[63] Rossell SL, David AS. Are semantic deficits in schizophrenia due to problems with access or storage? Schizophr Res. 2006;82(2–3):121–34. 10.1016/j.schres.2005.11.001 16386407

[64] Kerns JG, Berenbaum H. The relationship between formal thought disorder and executive functioning component processes. J Abnorm Psychol. 2003 8;112(3):339–52. 12943013 10.1037/0021-843x.112.3.339

[65] McGrath J. Ordering thoughts on thought disorder. Br J Psychiatry. 1991 3;158:307–16. 2036527 10.1192/bjp.158.3.307

[66] Liddle PF. Schizophrenic syndromes, cognitive performance and neurological dysfunction. Psychol Med. 1987;17(1):49–57. 3575577 10.1017/s0033291700012976

[67] Barrera A, McKenna PJ, Berrios GE. Formal thought disorder in schizophrenia: an executive or a semantic deficit? Psychol Med. 2005;35(1):121–32. 15842035 10.1017/s003329170400279x

[68] McKenna P, Oh TM. Schizophrenic Speech: making sense of bathroots and ponds that fall in doorways Cambridge: Cambridge University Press; 2005.

[69] O’Leary DS, Flaum M, Kesler ML, Flashman L a, Arndt S, Andreasen NC. Cognitive correlates of the negative, disorganized, and psychotic symptom dimensions of schizophrenia. J Neuropsychiatry Clin Neurosci. 2000;12(1):4–15. 10.1176/jnp.12.1.4 10678506

[70] Cuesta MJ, Peralta V. Cognitive disorders in the positive, negative, and disorganization syndromes of schizophrenia. Psychiatry Res. 1995;58(3):227–35. 8570778 10.1016/0165-1781(95)02712-6

[71] Basso MR, Nasrallah HA, Olson SC, Bornstein RA. Neuropsychological correlates of negative, disorganized and psychotic symptoms in schizophrenia. Schizophr Res. 1998;31(2–3):99–111. 9689714 10.1016/s0920-9964(98)00023-1

[72] Dibben CRM, Rice C, Laws K, McKenna PJ. Is executive impairment associated with schizophrenic syndromes? A meta-analysis. Psychol Med. 2008;39(3):381–92. 10.1017/S0033291708003887 18588741

[73] Brodbeck C, Pylkkänen L. Language in context: Characterizing the comprehension of referential expressions with MEG. Neuroimage. 2017;147:447–60. 10.1016/j.neuroimage.2016.12.006 27989776

[74] Egorova N, Shtyrov Y, Pulvermüller F. Brain basis of communicative actions in language. Neuroimage. 2016;125:857–67. 10.1016/j.neuroimage.2015.10.055 26505303 PMC4692511

[75] Peeters D, Snijders TM, Hagoort P, Özyürek A. Linking language to the visual world Neural correlates of comprehending verbal reference to objects through pointing and visual cues. Neuropsychologia. 2017;95:21–9. 10.1016/j.neuropsychologia.2016.12.004 27939189

[76] Kuperberg GR, Ditman T, Choi Perrachione A. When proactivity fails: An electrophysiological study of establishing reference in schizophrenia. Biol Psychiatry. 2017;3(1):77–87. 10.1016/j.bpsc.2017.09.007 PMC5801772 29397083

[77] Binder JR, Desai RH, Graves WW, Conant LL. Where is the semantic system? A critical review and meta-analysis of 120 functional neuroimaging studies. Cereb cortex. 2009 12;19(12):2767–96. 10.1093/cercor/bhp055 19329570 PMC2774390

[78] Cantiani C, Choudhury NA, Yu YH, Shafer VL, Schwartz RG, Benasich AA. From Sensory Perception to Lexical-Semantic Processing: An ERP Study in Non-Verbal Children with Autism. PLoS One. 2016;11(8):e0161637 10.1371/journal.pone.0161637 27560378 PMC4999236

[79] Ralph MAL, Jefferies E, Patterson K, Rogers TT. The neural and computational bases of semantic cognition. Nat Rev Neurosci. 2016;18(1):42–55. 10.1038/nrn.2016.150 27881854

