# Supplementary material for: Deficits in nominal reference identify thought disordered speech in a narrative production task
Source: PLoS One. 2018 Aug 7;13(8):e0201545. doi: 10.1371/journal.pone.0201545 (PMC6080774; doi:10.1371/journal.pone.0201545)
Supplement: S1 Appendix — NB: Examples of the first 7 variables illustrate anomalies. (DOCX) [file pone.0201545.s001.docx]

**Supplementary materials**

**Table 1: Variables, definitions and examples**

Variables 1-5 capture referential anomalies occurring in the respective NP type. Variables 6-7 involve lexical-level anomalies. Variables 8-10 are measures of syntactic complexity and integrity.

| **Variables** | **Definitions** | **Examples** |
| --- | --- | --- |
| 1. Pronoun | NPs without lexical content | *She* walked in the forest [beginning of the fairy tale, where we don’t know who is ‘she’] |
| 2. Noun | NPs with lexical content | *The girl* walked in the forest [beginning of the fairy tale, where we don’t know who is ‘the girl’] |
| 3. Definite | Lexical NPs containing determiners such as *the* or *this*, proper names, and pronouns, in their most standard uses where a specific referent is required. | *The girl* walked in the forest [where ‘a girl’ would be expected] |
| 4. Indefinite | Lexical NPs containing determiners such as *a* or *some*, or quantifiers like *nobody* or *someone*, or NPs without a determiner, in used where no specific referent is required. | The wolf chased the girl. *Someone* caught her. |
| 5. 3rdP | NP in the grammatical third Person. | Any of the above italicized examples. |
| 6. Paraphasia | Use of unexpected lexical nominal for a given referent. | Red Riding Hood walked into the *park* [instead of *forest*] |
| 7. Semantic selectional restriction | Impossible combinations of phrases based on their lexical meaning. | The girl *drank* the *wolf*. |
| 8. Complement clause | Clause embedded in another, forming the grammatical object of a verb such as *think* or *say*. | She thinks *that the wolf is her grandmother* |
| 9. Formal grammatical error | Violation of grammatical well-formedness conditions. | The girl *meet* wolf [Agreement violation and missing determiner] |
| 10. Number of dependents per utterance | Number of units of discourse grammatically depending on a given head. | *The girl* visited *the grandmother* (two dependents). |
